# Detection of Seasonal Variation in *Aloe* Polysaccharides Using Carbohydrate Detecting Microarrays

**DOI:** 10.3389/fpls.2019.00512

**Published:** 2019-05-14

**Authors:** Louise Isager Ahl, Narjes Al-Husseini, Sara Al-Helle, Dan Staerk, Olwen M. Grace, William G. T. Willats, Jozef Mravec, Bodil Jørgensen, Nina Rønsted

**Affiliations:** ^1^Natural History Museum of Denmark, University of Copenhagen, Copenhagen, Denmark; ^2^Department of Drug Design and Pharmacology, University of Copenhagen, Copenhagen, Denmark; ^3^Comparative Plant and Fungal Biology, Royal Botanic Gardens Kew, Richmond, United Kingdom; ^4^School of Natural and Environmental Sciences, Newcastle University, Newcastle upon Tyne, United Kingdom; ^5^Department of Plant and Environmental Sciences, University of Copenhagen, Frederiksberg, Denmark

**Keywords:** *Aloe*, authentication, carbohydrate detecting microarrays, plant cell walls, polysaccharides, seasonal variation, succulent tissue

## Abstract

*Aloe vera* gel is a globally popular natural product used for the treatment of skin conditions. Its useful properties are attributed to the presence of bioactive polysaccharides. Nearly 25% of the 600 species in the genus *Aloe* are used locally in traditional medicine, indicating that the bioactive components in *Aloe vera* may be common across the genus *Aloe*. The complexity of the polysaccharides has hindered development of relevant assays for authentication of *Aloe* products. Carbohydrate detecting microarrays have recently been suggested as a method for profiling *Aloe* polysaccharide composition. The aim of this study was to use carbohydrate detecting microarrays to investigate the seasonal variation in the polysaccharide composition of two medicinal and two non-medicinal *Aloe* species over the course of a year. Microscopy was used to explore where in the cells the bioactive polysaccharides are present and predict their functional role in the cell wall structure. The carbohydrate detecting microarrays analyses showed distinctive differences in the polysaccharide composition between the different species and carbohydrate detecting microarrays therefore has potential as a complementary screening method directly targeting the presence and composition of relevant polysaccharides. The results also show changes in the polysaccharide composition over the year within the investigated species, which may be of importance for commercial growing in optimizing harvest times to obtain higher yield of relevant polysaccharides.

## Introduction

The succulent *Aloe vera* L. leaf tissue is a natural product used globally in a wide range of household commodities (Grace et al., 2015). By the end of 2016, *Aloe vera* leaf tissue had reached a revenue of US$ 1.6 billion and it is estimated that the revenue will exceed US$ 3.3 billion by 2026 (Future Market Insights, 2016). The succulent inner leaf tissue, the gel, is a polysaccharide rich matrix containing high amounts of mannan (polymannose), which enables the tissue to hold larger amounts of water (Reynolds and Dweck, 1999; Ni et al., 2004; Grace et al., 2015). The genus *Aloe*, to which *Aloe vera* belongs, contains more than 500 different species out of which at least 25% are used medicinally mainly by indigenous communities in the areas where they occur naturally (Grace, 2011).

From the *Aloe* leaves, two different medicinal products can be derived – the excudate and the gel. The often yellow and bitter exudate comes from aloitic cells (specialized cells in relation to the vascular bundles, that excrete af mixture of compounds used for medicinal purposes (Reynolds, 2004) in the outer leaf mesophyll, and contains a range of compounds used as purgative (Grace et al., 2009). The colorless polysaccharide-rich gel from the inner leaf is used topically for treatment of wounds, minor burns, and skin irritation or internally for a range of different applications (Grindlay and Reynolds, 1986; Reynolds and Dweck, 1999; Hamman, 2008; Grace et al., 2009). Due to the complexity of the polysaccharides, the composition and bioactivity of *Aloe* gel is not well understood, and there is a lack of useful methods for analysis and authentication (Bozzi et al., 2007; Grace and Rønsted, 2017).

The plant cell wall is an insoluble entity composed almost entirely of complex polysaccharides arranged in an intricate matrix (Cosgrove, 2005; Albersheim, 2011). The main non-cellulosic polysaccharides in *Aloe* inner leaf mesophyll are hemicelluloses and pectins. Hemicelluloses cover a range of different polysaccharides with xyloglucans usually being the principal ones (Albersheim, 2011; Pedersen et al., 2012). Another hemicellulose mannan, and in particular an acetylated form of it, have been of particular interest in relation to *Aloe* research as it is considered the most likely bioactive component in the gels (Reynolds and Dweck, 1999; Talmadge et al., 2004; Simões et al., 2012). Plant cell wall polysaccharides are traditionally investigated indirectly using monosaccharide analyses (Albersheim, 2011; Grace et al., 2013), but by the complete break-down of the plant cell wall, information is inevitably lost about the tertiary structure and chemical construction of the polymers, why development of methods targeting polysaccharides or at least oligosaccharides have been highly sought after (Fangel et al., 2012; Krešimir et al., 2017).

The ability to analyze and distinguish between polysaccharide compositions in different plant tissues, between different batches, and between species are especially important in plants containing bioactive polysaccharides used for medicinal purposes like the acetylated mannan of *Aloe vera* (Femenia et al., 1999; Ahl et al., 2018; Minjares-Fuentes et al., 2018). Mannan is not only a common plant cell wall polysaccharide, but it is also often found in tissues related to water storage (Stancato et al., 2001). The acetylated mannan (polymannose) from *Aloe vera* has been linked to induced tissue repair in humans (Reynolds and Dweck, 1999; Xing et al., 2014; Thunyakitpisal et al., 2017), whereas a de-acetylation of mannan have been shown to result in a loss of bioactivity (Chokboribal et al., 2015).

Polysaccharide and phenolic compound contents are expected to vary with age of the plant, between batches, and with season and rainfall or water availability (Hu et al., 2003; Beppu et al., 2006; Cristiano et al., 2016). Harvesting and subsequent processing including drying of *Aloe* gel can also influence the content and composition of bioactive compounds including causing de-acetylation of mannan polymers (Minjares-Fuentes et al., 2016; Sriariyakul et al., 2016).

The efficacy and safety of herbal products can be compromised through accidental adulteration, misidentification and deliberate contamination, which can lead to lack of the desired effect at best, or severe side effects due to the presence of toxic compounds in worst case scenarios (Ernst, 2004; van Breemen and Farnsworth, 2008; Gilbert, 2011; Saslis-Lagoudakis et al., 2015). To ensure the efficacy and safety of herbal products, their qualitative and quantitative composition are regulated by international and national monographs such as the European Pharmacopeia by the European Directorate for the Quality of Medicines and Healthcare (EDQM, 2016), which presents a series of monographs for herbal products, including recommended tests for identification and quality of the plant species included in these products.

Two bulk *Aloe* herbal products are included in the European Pharmacopeia (EDQM, 2016), namely *Aloe barbadensis* (a synonym of the accepted name, *Aloe vera* L.), and *Aloe capensis* (a synonym of the accepted name, *Aloe ferox* Mill.), but both are based on the detection of hydroxyanthracene derivatives in the juice (exudate). A World Health Organization [WHO] (1999) monograph is available on *Aloe vera* gel recommending a chromatographic assay (t’Hart et al., 1989; World Health Organization [WHO], 1999), but no quantitative requirements of content has been proposed.

Considering the global use and appraisal of *Aloe vera* gel and its acclaimed beneficial effects, there is an urgent need for establishing reliable, and relevant authentication methods. In addition to ensuring the safety and efficacy of *Aloe* herbal products, an authentication method can also be used to assist in control of illegal harvesting and trade. All *Aloe* species except *Aloe vera* are prohibited from trade under the Convention on International Trade in Endangered Species as described in appendix II (CITES, 2017).

Due to the complexity of the polysaccharides, no efficient standard method exists for neither qualitative nor quantitative authentication of polysaccharide composition in *Aloe* herbal products (Grace et al., 2013; Minjares-Fuentes et al., 2018). Full structural identification of polysaccharides can currently only be achieved through a complex combination of spectroscopic techniques (Simões et al., 2012; Shi et al., 2018). However, a number of indirect methods exist, such as ^1^H-NMR spectroscopy, which can be used to verify the presence of specific structural groups, such as the acetyl groups of the acetylated mannan (Bozzi et al., 2007; Campestrini et al., 2013).

Structure–activity relationships suggest that monosaccharide composition and branching patterns play an important role in the bioactivity of plant polysaccharides (Paulsen and Barsett, 2005). As a proxy, the constituent monosaccharides have therefore been suggested as a tool for authenticating *Aloe*-based products (O’Brian et al., 2011; Minjares-Fuentes et al., 2018). Several analytical techniques are in use including colorimetric and spectrophotometric fingerprinting methods, and chromatographic methods, which can efficiently separate, identify, and quantify the monosaccharides (t’Hart et al., 1989; Eberendu et al., 2005; Nazeam et al., 2017; Zhang et al., 2018). However, little is known about the relationship between polysaccharide composition and therapeutic value of the leaf mesophyll in *Aloe*, and it is recommended that future authentication focus on developing methods targeting the polysaccharides (Grace et al., 2013).

Carbohydrate detecting microarrays (Moller et al., 2007) have been proposed as a possible method for qualitative comparison of polysaccharide composition between *Aloe* species and in *Aloe* herbal products (Ahl et al., 2018). Carbohydrate detecting microarrays is a high-throughput method allowing for the simultaneous investigation of numerous samples at the same time. However, carbohydrate microarrays are limited by what antibodies are available and the effectiveness of extractions and immobilization. The most optimal use of the method in relation to authentication is as a complementary screening tool prior to analyses like ^1^H-NMR spectrometry analysis for more in-depth knowledge of the present *Aloe* compounds (Campestrini et al., 2013; Minjares-Fuentes et al., 2018). For the purpose of obtaining quantitative data, GC-MS profiling of monosaccharides is also still a useful method (Grace et al., 2013).

The aim of the present study was to use carbohydrate detecting microarrays to investigate the seasonal variation in the polysaccharide composition of two medicinal and two non-medicinal aloes over the course of a year. Microarray profiling was complemented by microscopy to understand where in the cells the bioactive polysaccharides are present.

## Materials and Methods

### Plant Material

Four species were chosen for this study to represent medicinal and or non-medicinal usage, but also based on their growth form, geographical distribution, and leaf size (Table 1). *Aloe vera* is a short-stemmed species growing in large clumps, and is probably native to the Arabian Peninsula (Grace et al., 2015). *Aloe arborescens* is a widespread species in the southern part of the African continent. The two medicinal aloes are very different in terms of habit, growth form and distribution, with *A. arborescens* growing up to 3 m in height compared to *A. vera* being a maximum of 1 m tall. Both non-medicinal species selected for this study are native to Madagascar, with *A. decaryi* being a narrow endemic growing in a pendulous or sprawling habit in thickets near sea level. *Aloe vaombe*, on the other hand is a widespread tree growing up to 5 m tall at altitudes of 50–1200 m (Carter et al., 2011).

**Table 1:** Biogeographical, morphological, and usage of the selected *Aloe* species found in the wild.

		Plant name			
		*Aloe decaryi* Guillaumine	*Aloe vaombe* Decorse & Poiss	*Aloe vera* L. Burm.f.	*Aloe arborescens* Miller
*In situ*	**Region (s)**	Madagascan	Madagascan	Only in cultivation	Southern African Zambezian
	**Country**	Madagascar	Madagascar	Only in cultivation	South Africa (L, M, KN, EC, WC) Swaziland Malawi MozambiqueZimbabwe
	Distribution	Limited – island	Widespread – island	Naturalised	Widespread
	Ecological adaptations	Thick coastal scrub	Dry thorn-bush	NA	Rocky slopes, sometimes dense bush
	Altitude min (m)	Sea level	50	NA	Sea level
	Altitude max (m)	Sea level	1200	NA	2800
	Type	Pendulous/sprawling	Tree	Stemless - large clumps, few-branched	Tree
	Fresh sap	NA	Deep purple	Yellow	NA
	Dry sap	NA	Deep purple	Yellow	NA
	Lenght (cm) leaves min	15	80	40	50
	Lenght (cm) leaves max	19	100	60	60
	Whith (cm) leaves min	0.8	15	6	5
	Whith (cm) leaves min	1.2	20	7	7
	Leaf color	Uniformly dull green	Dull green	Grey-green with brownish tinge	Dull green to grey-green
	Teeth	Present	Present	Present	Present
	Flower color	Rose-red to scarlet	Bright crimson	Yellow	Scarlet
	Usage	non-medicinal	non-medicinal	medicinal	medicinal
	Year scientifically identified	1941	1912	1768	1768
Greenhouse	Voucher details	Ahl P1977-5375	Ahl S1985-0546	Ahl P1991-5289	Ahl 1997-0785
	Spring watering	1–2 times per week	1–2 times per week	1–2 times per week	1–2 times per week
	Summer watering	2–3 times per week	2–3 times per week	2–3 times per week	2–3 times per week
	Fall watering	1–2 times per week	1–2 times per week	1–2 times per week	1–2 times per week
	Winter watering	1–2 times per week	1–2 times per week	Every 3rd week	Every 3rd week
	Extra light in winter	Yes	Yes	No	No
	Temperature (°C)	18–22	18–22	13–17	13–17
	Fertilizer	NPK standard	NPK standard	NPK standard	NPK standard

*The cultivated species were grown in two different greenhouses depending on their natural habitat. *Aloe arborescens* and *Aloe vera* did not get additional light during the winter months, but *Aloe vaombe* and *Aloe decaryi* did, as they grow mainly in the winter months. Watering was generally done according to the directions above, but if a time period had been particularly sunny watering was increased and if the period had been more cloudly watering was decreased. Temperature were adjusted to be within the listed ranges. Vouchers are deposited in Herbarium C, National History Museum of Denmark, University of Copenhagen, Denmark.*

Plant material was sampled from the living collections of the Botanical Garden, Natural History Museum of Denmark, University of Copenhagen, Denmark, and vouchers are deposited in Herbarium C (Table 1).

Plants of the four species were mature (+20 years old) when sampled and were grown under glass in conditions mimicking the daylight changes and water availability of the region they come from (Table 1). Samples were collected in triplicates for each species once a month from June 26th, 2017 to June 25th, 2018. Seasonality are expressed as northern hemisphere spring (March–May), summer (June–August), autumn (September–November), and winter (December–February) for the greenhouse-grown material, although the natural habitat of most aloes is in the southern hemisphere (Table 1). Two different types of fresh samples were taken at each collection point. To reduce the risk of contamination with phenolic compounds, which can bind and lead to masking of epitopes in the carbohydrate detecting microarrays, only the inner leaf mesophyll was carefully collected for carbohydrate detecting microarray analysis. Sections including epidermis were collected for microscopy investigations.

Ph. Eur. reference material for *Aloe vera* (product number 103504) and *A. capensis* (product number 203304) was obtained from Alfred Galke, Bad Grund, Germany, and was used as a standard (EDQM, 2016).

### Microarray Profiling of Polysaccharides

**FIGURE 1:**
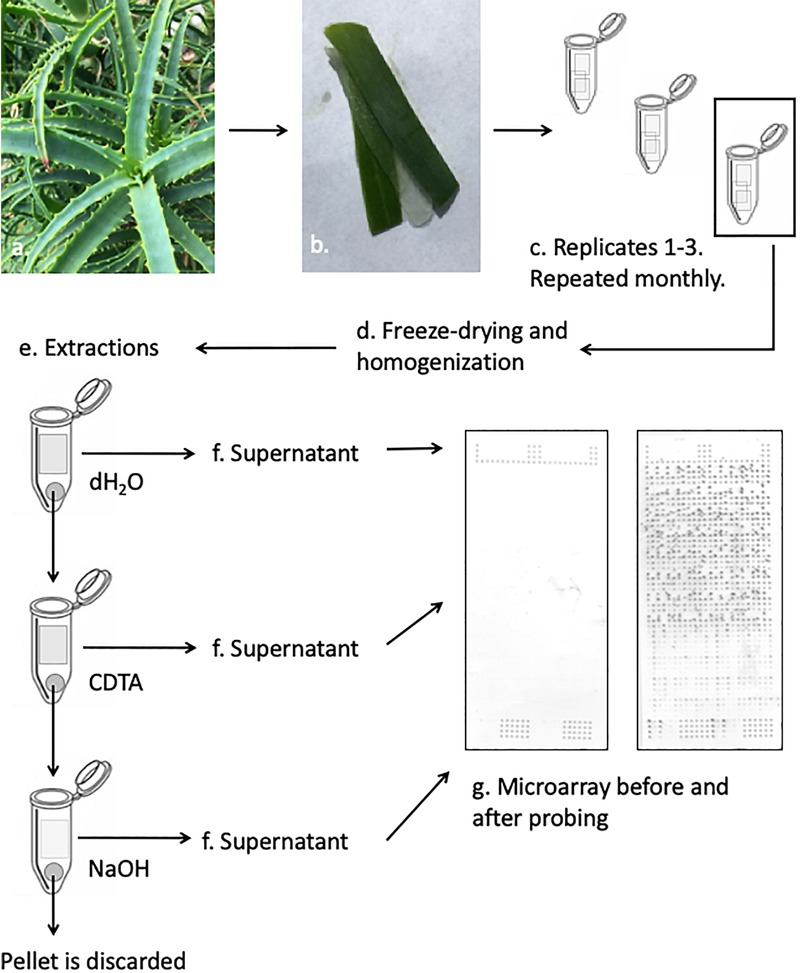
Schematic presentation of the CoMPP method illustrated with *Aloe arborescens*. Species are sampled in triplicates and prepared for extractions. The supernatant from each sequential extraction step is printed separately in technical replicates on nitrocellulose membranes. The printed microarrays are then probed with a selection of molecular probes.

Polysaccharide data was obtained by following the protocol described by Ahl et al. (2018), which in turn was modified from that of Moller et al. (2007) to accommodate the unique properties of the *Aloe* tissue (Figure 1). The succulent inner leaf mesophyll was collected in three biological replicates from the four selected *Aloe* species each month for a full year. The tissue was carefully excised from mature leaves, and immediately placed in labeled Falcon tubes (Corning, New York, United States) before they were snap frozen in liquid nitrogen. The collected samples were kept at –20°C for 24 h before they were freeze dried, weighed, and milled prior to extractions. Samples of approximately 5 mg were weighed to 1 decimal accuracy from each biological replicate and placed in Corning 8-strip cluster tubes (Merck Life Science, Darmstadt, Germany). Samples were homogenized in a Tissuelyser II (Gentec Biosciences, Columbia) using glass beads prior to extractions.

Extractions were carried out in three-step sequential series and for each sample, the extractant volume was adjusted to accommodate the exact weight of each sample reaching a ratio of 10 mg sample to 300 μL extraction solvent. The extraction series is based on the work by Moller et al. (2007) and adjusted according to Ahl et al. (2018). The glass beads used for homogenization were kept in the tube to enhance the extraction of polysaccharides during the sequential steps. The following solvents were used: dH_2_O – targeting primarily soluble unbound or loosely bound polysaccharides including mannans, 50 mM CDTA (*trans*-1,2-diaminocyclohexane-*N,N,N′,N′*-tetraacetic acid monohydrate, pH 7.5, Merck Life Science, Darmstadt, Germany) – targeting primarily pectins and some hemicelluloses, and finally 4 mM NaOH – targeting primarily hemicelluloses. For all three extraction steps samples were shaken in a Tissuelyzer at 27 s^-1^ for 2 min before the speed was reduced to 6 s^-1^ for 2 h. All extractions were carried out at room temperature. After the extractions, samples were centrifuged at 4000 RPM (Thermo Fisher Scientific, Waltham, MA, United States) for 10 min before the supernatant was carefully removed and transferred to a labeled 0.5 mL Eppendorf tube (Eppendorf, Hamburg, Germany). Extractions were carried out on the pellet, and extracts were kept at 4°C during the subsequent extractions to minimize degradation.

Once extractions were done for all samples, fourfold dilution series were made for each sample in a 384-well microtiter plate (Merck Life Science, Darmstadt, Germany). Dilutions were made using Arrayjet buffer (55.2% glycerol, 44% water, 0.8% Triton X-100). The 384-well microplates with the diluted extracts were centrifuged at 3000 RPM (Thermo-Fisher Scientific, Waltham, MA, United States) for 10 min before they were printed on a 0.45 μm nitrocellulose membrane (Whatman, Maidstone, United Kingdom) using an Arrayjet Sprint (Arrayjet, Edinburgh, United Kingdom) piezoelectric robotic printer. For each sample, the dilution series was printed in four technical replicates on each microarray, to yield a total of 48 spots per plant specimen per harvest (16 spots per extraction step). The three biological replicates were extracted and printed on three separate days using the approach described above.

**FIGURE 2:**
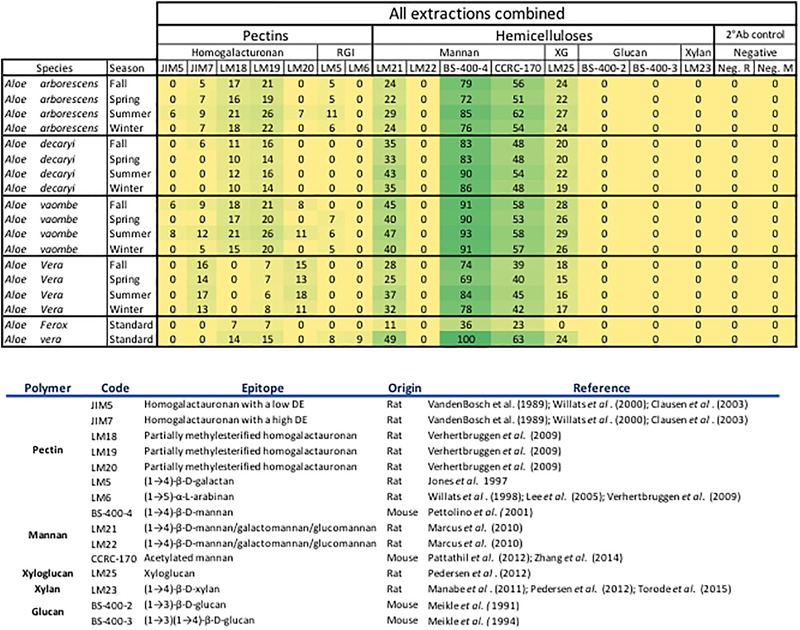
All extractions are summarized per season. The Ph. Eur. Material extractions (standards) are also summarized for each species. All tested antibodies are included. Included antibodies and their target epitope as well as the origin of the antibody and where their bindings have been described is listed below the heatmap. DE, degree of esterification; RGl, rhamno-galacturonan; KG, kyloglycan; Neg. R, anti-rat; Neg. M, anti-mouse. The highest mean value of the entire dataset was assigned the value of 100%, and the remainder of the data were adjusted accordingly and normalized with a 5% cut off (represented with a zero – “0”).

Fifteen primary monoclonal antibodies were selected to cover as many types of different pectic and hemicellulotic polysaccharide epitopes as possible (Figure 2). The primary antibodies were paired with either alkaline phosphatase conjugated anti-rat or anti-mouse as secondary antibody (Merck Life Science, Darmstadt, Germany) depending on the origin of the primary antibody. The printed arrays from all three identical extraction rounds were developed, quantified and analyzed simultaneously following the procedures described by Ahl et al. (2018). The final tally of arrays developed for this study accounts to 47 arrays: 1 for each antibody and extraction round, plus two for negative controls of the secondary antibodies.

For the data analysis averages were calculated using both the dilution series for each sample and the array triplicates (total of 48 data points per sample). The full data set was visualized in a heatmap format with all antibodies and their binding shown in the Figure 2. The highest mean value of the entire dataset was assigned the value of 100%, and the remainder of the data were adjusted accordingly and normalized with a 5% cut off (represented with a zero – “0”). All data analyses were carried out in Microsoft Excel for Mac, version 16.16.4 (181110), 2018.

### Microscopy

The microscopy work was done on samples from the summer collection in August 2017.

**FIGURE 3:**
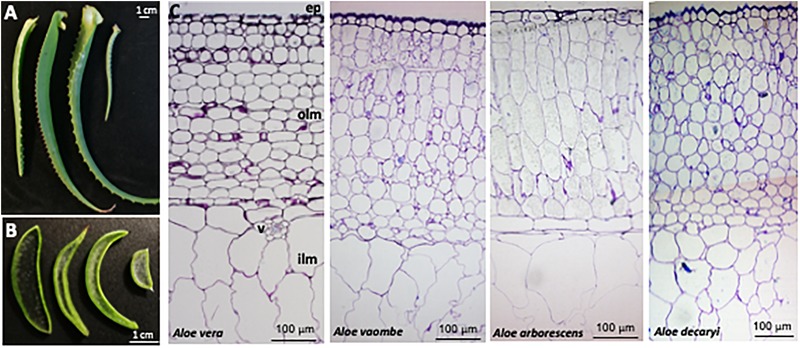
Anatomy of four *Aloe* species, **(A)** Leaves and **(B)** cross-sections from left to right of *Aloe vera, A. vaombe, A. arborescens* and *A. decaryi*. **(C)** Cross-sections from left to right of *Aloe vera, A. voombe, A. arborescens* and *A. decaryi* stained with Toluidine blue. Marked are ep, epidermis; olm, outer leaf mesophyll; v, vasculature; ilm, inner leaf mesophyll.

Sections from all four *Aloe* species were also stained with 1% Toluidine blue for 10 min, washed twice in distilled water and mounted on glass slides under a coverslip. Images were taken on Olympus BX41 microscope with a mounted Olympus ColorView I camera (Figure 3).

Tissue pieces of approximately 3 mm in diameter were excised from the sampled material and fixed for 30 min in 4% formaldehyde prepared from paraformaldehyde in phosphate-buffered saline (PBS). Sections were washed twice in PBS, before they were dehydrated in a series of methanol:water solutions until reaching a final concentration of 100% methanol. The methanol was then substituted with a methanol:LR White resin mixture (1:1) for 8–10 h. Sections were then transferred to a pure LR resin overnight. The specimens were organized in gelatine capsules filled with pure LR resin. The final polymerization was performed overnight in a 60°C oven. 1 μm-thick sections were made from each species using a Leica EM-UC7 ultramicrotome (Leica, Roskilde, Denmark) and glass knives, and subsequently adhered on Superfrost Slides (Thermo Scientific, Roskilde, Denmark) in a drop of water at 60°C.

**FIGURE 4:**
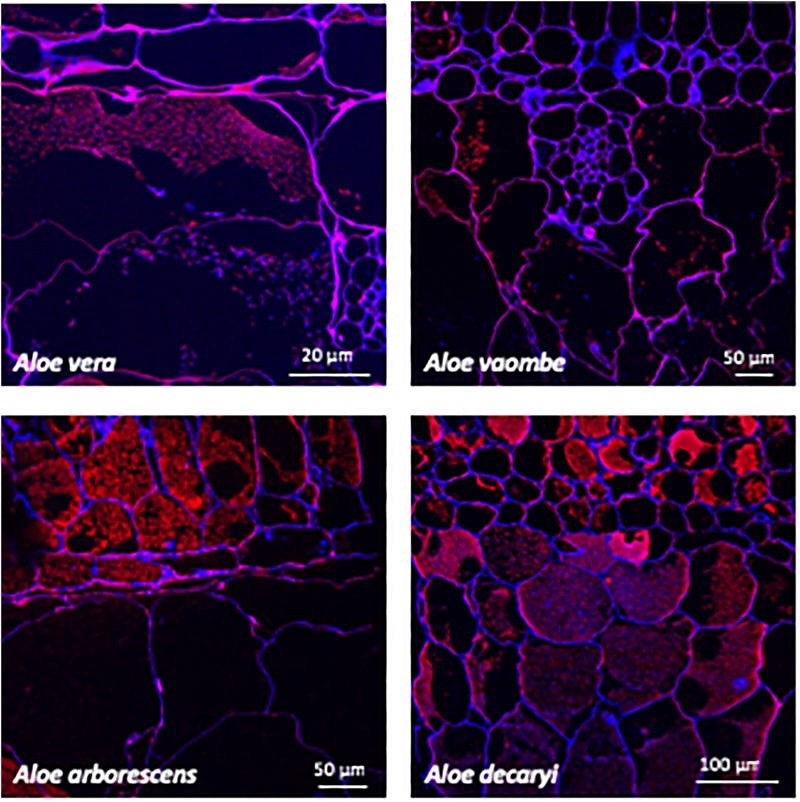
Immunolabeling using the mannan specific monoclonal antibody BS-400-4 (red channel). Overlay with CalcofluorWhite (blue channel) counterstain to visualize cell walls.

**FIGURE 5:**
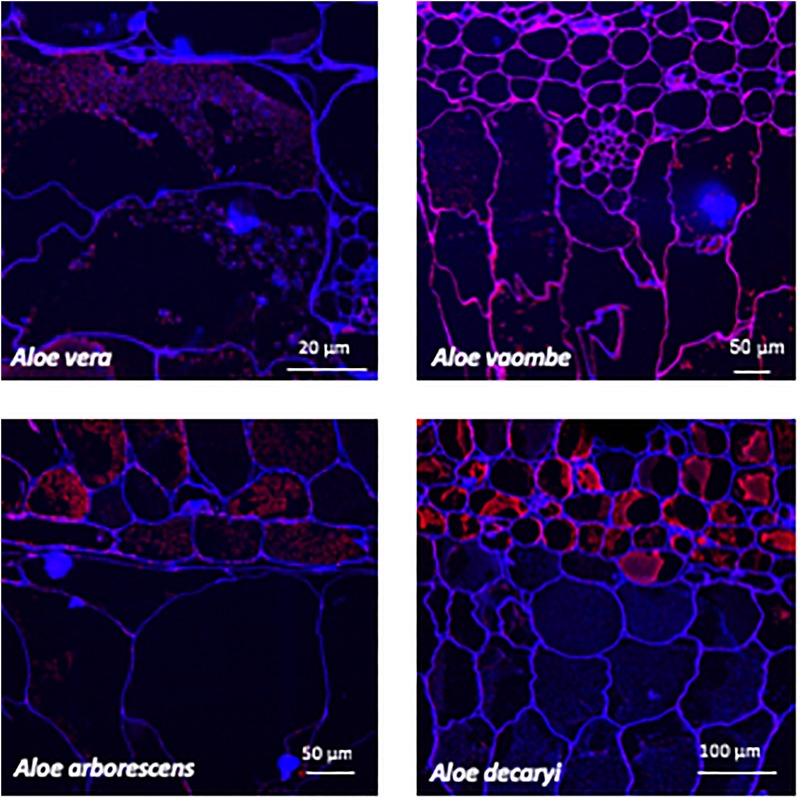
Immunolabeling using the mannan specific monoclonal antibody LM21 (red channel). Overlay with Calcofluor White (blue channel) counterstain to visualize cell walls.

Immunolocalization of different polysaccharides in resin sections was performed following the procedure described by Mravec et al. (2017) using the antibodies BS-400-4 and LM21 (Pettolino et al., 2001; Marcus et al., 2010). In short, leaf sections were placed on individual glass microscope slides, and a hydrophobic circle was drawn around each section with a PAP pen (Merck Life Science, Darmstadt, Germany). Sections were then blocked with a 5% milk powder and PBS solution for 15 min, before probing with the monoclonal antibodies (Figure 4, 5) for 1 h. Antibodies were diluted 1:10 in a 5% milk powder and PBS solution. Sections were then washed twice with 5% milk powder in PBS solution, before probing with secondary antibodies. The secondary antibodies used were either anti-rat or anti-mouse conjugated to Alexa Fluor 555 (Invitrogen, Roskilde, Denmark) at 1:300 dilution in 3% bovine serum albumin (BSA) in PBS. Leaf sections were then washed three times in PBS, and counterstained with Calcofluor White (Merck Life Science, Darmstadt, Germany) at 0.1 mg/ml concentration for 10 min. Finally, the leaf sections were washed one last time before being mounted in CitiFluor, an antifading reagent (Agar Scientific, Essex, United Kingdom).

The fluorescently labeled samples were scanned using a Leica SP5 confocal laser scanning microscope equipped with UV diode (405 nm), Ar (488 nm), and HeNe (543 nm) lasers at either 20X or 63X water objectives. Pictures were processed with GIMP2 software for color enhancement and contrast. Control samples were treated equally for comparison.

## Results

### Aloe Inner Leaf Mesophyll Structure and Localization of Polysaccharides

Distinctive morphological differences between the four different *Aloe* leaves investigated for this study were observed (Figure 3). The amount of inner leaf mesophyll varied from a thin layer in *A. vaombe* to a thicker many-celled layer in *A. vera*. The micrographic observation of toluidine blue stained resin sections showed the basic anatomical structure is conserved between the *Aloe* species. Below epidermis, is the outer mesophyll made of app. 15 layers of round or slightly elongated parenchymatic cells with size of 40–100 μm in diameter followed by the inner mesophyll of enlarged water storage cells reaching 300 μm in diameter. These observations showed that the overall thickness of the *Aloe* leaves is largely determined by the thickness of the inner mesophyll.

Two mannan-specific antibodies (BS-400-4 and LM21) were used to investigate the localization of mannans in the mesophyll layers. The exact specificity of the mannan-binding antibody BS-400-4 is (1→4)-β-mannan/galacto-(1→4)-β-mannan (Pettolino et al., 2001). The probed micrographs (Figure 4, 5) show that that the majority of mannan is found in the cytosol inside the cells, and in very different amounts depending on the species. In *A. vaombe* the mannan seems to be almost entirely embedded in the wall. The mannan polymers do not seem to form a continuous entity, but rather appear as granules. In *A. decaryi* the mannan granules fill up almost the entire inside of the cells, whereas there were distinct vacuoles in the gatherings of mannan polymers in *A. vera* and *A. arborescens*. Additionally, in *A. arborescens* it seems like mannan is primarily present in the outer mesophyll and only present in thin bands around the edges in the inner mesophyll. The mannan-specific antibody LM21 binds β-(1→4)-manno-oligosaccharides from DP2 to DP5, but it also displays a wider recognition including mannan, glucomannan and galactomannan polysaccharides (Marcus et al., 2010). Based on the histological micrographs (Figure 5) from the four *Aloe* species there appear to be very little mannan present in the mesophyll when LM21 is used to detect it. Again, in *A. vaombe*, the mannan seems to be almost embedded in the wall, whereas the distribution resembles the binding pattern of BS-400-4 more in the remaining three species although in a lower concentration. In *A. vera, A. arborescens*, and *A. decaryi* the mannan recognized by LM21 also seems to be granulated rather than a dense sheet (Figure 4).

### Polysaccharide Profile Variation Between Species

**FIGURE 6:**
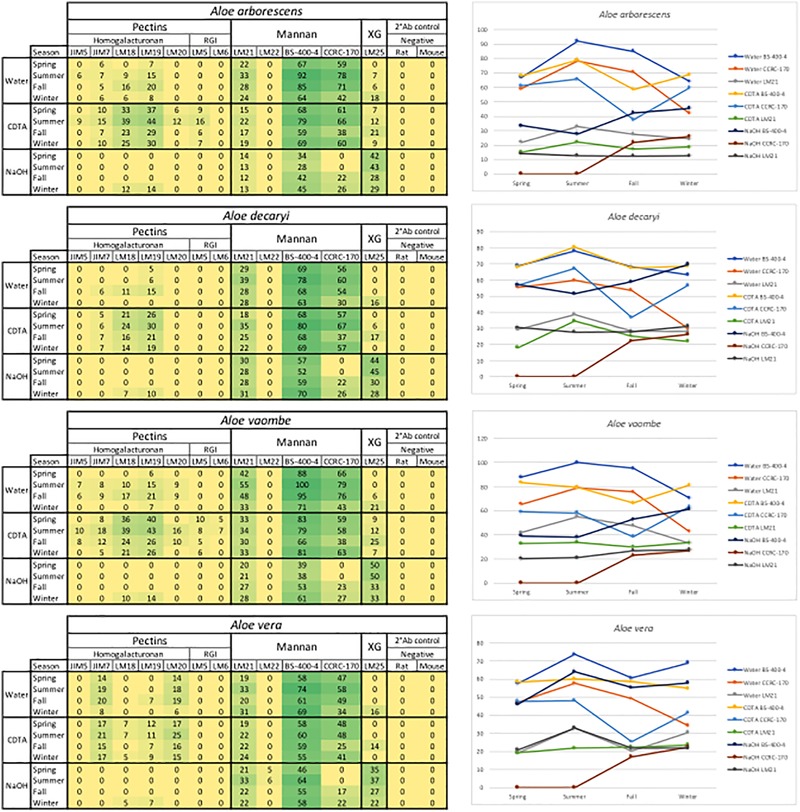
Species specific heatmaps and graphs showing the changes in mannan over time in the three sequential extractions. RGI, rhamno-galacturonan; XG, xyloglycan; Meg. R, anti-rat; Neg. M, anti-mouse. The highest mean value of the entire dataset was assigned the value of 100%, and the remainder of the data were adjusted accordingly and normalized with a 5% cut off (represented with a zero – “0”).

Binding studies of 15 primary monoclonal antibodies representing different pectic and hemicellulotic polysaccharide epitopes show differences between the four *Aloe* species in polysaccharide compositions of their inner leaf mesophyll (Figure 2, 6). As expected, the sequential extraction series resulted in the extraction of a mixture of polysaccharides. Figure 2 presents a summary of the pooled serial extractions for each species and each antibody, in order to investigate if the total amounts of polysaccharides change over the course of a year, whereas detailed results for each of the three extraction solvents (H_2_O, CDTA, and NaOH) are shown in the Supplementary Material. In Figure 6, species-specific heatmaps including only the antibodies that recognized epitopes in the material are depicted along with graphs showing the changes in mannan epitopes over time.

For all species mannan and xyloglycan epitopes were detected, although in various amounts. *A. vaombe* contained the highest amounts of mannan in the water extractions as detected by all three mannan-specific antibodies (LM21, BS-400-4, and CCRC-170) seen in Figure 6 (Pettolino et al., 2001; Marcus et al., 2010; Pattathil et al., 2012; Zhang et al., 2014). For all species, the CCRC-170, binding acetylated mannan, the signal completely disappears in the spring and summer samples during the NaOH extraction, but re-appear in lower concentrations during the fall and winter.

The most distinct changes between species are found in the pectin profiles and this has also been observed in other studies (Ahl et al., 2018). In relation to specific antibodies, only *A. vaombe* and *A. arborescens* show binding from JIM5, targeting low methylated homogalacturonan – the backbone of the pectin polymer (Vandenbosch et al., 1989; Willats et al., 2000; Clausen et al., 2003). High-methylated homogalacturonan (JIM7), expressed by the binding of JIM7, is primarily released from the matrix in the CDTA extraction, but only in very low amounts from *A. decaryi*. Slightly more is released from *A. arborescens* and *A. vaombe*. The highest amounts of JIM7 is released from *A. vera* reaching almost double the amount when all seasons and extractions are combined (Figure 2; Vandenbosch et al., 1989; Willats et al., 2000; Clausen et al., 2003). Three different antibodies are detecting partially methylated homogalacturonan – LM18, LM19, and LM20 (Verhertbruggen et al., 2009). Despite being described as binding to the same type of epitope there are clear differences in the binding patterns of the three antibodies. There is binding for LM18 and LM19 in all selected species, but LM20 does not show any binding to *A. decaryi* and also has a very low binding to *A. vaombe* and *A. arborescens*, but then binds strongly to *A. vera* in the same pattern as JIM7 did. In terms of LM18 and LM19, *A. vera* is the species with the lowest binding with amounts hardly above the background cut-off. The three remaining species all express strong binding to both LM18 and LM19 with noticeable differences between the seasons. For all three species relative amounts of the polysaccharides are almost doubled in the spring and summer periods compared to fall and winter.

In this study two antibodies targeting galactan and arabinan epitopes on rhamno-galacturonan, a pectin side-chain, was included – LM5 and LM6 (Jones et al., 1997; Willats et al., 1998; Lee et al., 2005). LM6 only bound to *A. vaombe* in the CDTA extraction of the spring and summer periods, but very weakly. Similarly, LM5 only showed binding in the CDTA extractions of *A. vaombe* and *A. arborescens*. Again, the binding showed low amounts of galactan with the highest values found in the spring and summer periods. Whereas seasonal differences were clearly detectable in the different extraction steps (Figure 6 and Supplementary Material), the combined heatmap (Figure 2) shows that even though changes do occur over a 12-month period they are much subtler when considering the pooled extracts. The summer amounts are still the highest for almost all antibodies and all species. The differences between the species are still clearly visible even when extraction data is pooled (Figure 2).

## Discussion

### Organization of Mannans in the Succulent Tissue of Aloes

The microscopy work was done to determine the placement of the polysaccharides within the succulent tissue and determine the differences and similarities between the four species – *A. arborescens, A. decaryi, A. vaombe*, and *A. vera*. The microscopy work has overall corroborated the carbohydrate detecting microarrays results both in terms of species differences and the localization of specific polymers recognized by the same set of antibodies as were used for the carbohydrate detecting microarrays. However, on the histological micrographs, *A. vaombe* appeared to contain a low amount of mannan based on the detection of LM21 and BS-400-4 and only in the cell wall, whereas the carbohydrate detecting microarrays analysis of the comparable summer samples showed *A. vaombe* to be the one investigated species containing the most of both epitopes. In the microscopy study the focus was on the bioactive polysaccharide mannan using the antibodies BS-400-4 and LM21. Both anti-mannan antibodies detected the polymers in all species, but in *A. arborescens*, the signal from both antibodies BS-400-4 and LM21 was more pronounced in the outer mesophyll cells than in the inner leaf mesophyll (Figure 4, 5, respectively). This could indicate that the outer cell layers are more used for storage than the inner most cells are, assuming mannans function as storage polymers (Stancato et al., 2001). In *A. vaombe* the signals from the mannan recognizing antibodies were generally weaker, and the mannan appeared to be embedded in the cell wall with only very low amounts of the polysaccharide located in the cytosol. In terms of mannan amounts and distribution based on the histological micrographs, *A. vera* and *A. decaryi* seem to be containing the highest amounts of mannan, suggesting *A. decaryi* potentially could also be a source of medicinally relevant mannans. *A. arborescens* also contained larger amounts of mannan, but not throughout the mesophyll as did *A. vera* and *A. decaryi*. The extensive medicinal use of *A. arborescens* indicates that it is the quantity rather than the specific localization of the polysaccharides in the mesophyll that determines the medicinal quality of the mannan.

### Structural Function of *Aloe* Polysaccharides in the Cell Wall

A very general description of a plant cell wall is based on a scaffold of linear cellulose strands bound together by an array of hemicelluloses embedded in a pectin matrix (Cosgrove, 2005; Albersheim, 2011). The main non-cellulosic polysaccharides detected by carbohydrate detecting microarrays in *Aloe* inner leaf mesophyll are pectins and two kinds of hemicelluloses – mannans and xyloglucan. Whereas the acetylated mannan is interesting from a medicinal point of view, from a plant cell wall perspective, the xyloglucan is likely to be the primary one binding the cellulose strands together as the mannan seems to be present in granulates more than as a flat sheet when looking at the histological micrographs. A large concentration of mannan was released in the water extraction also suggesting that these polymers are very loosely bound in the matrix, as tightly bound hemicelluloses would normally be expected to require NaOH for bulk release (Hansen et al., 2014). The release of xyloglucan in the NaOH extraction thus further supports the idea that this polysaccharide is more tightly bound in the cell wall participating in the general scaffold together with cellulose. Neither xylan nor glucans were detected in the carbohydrate detecting microarrays analyses (seen by the lack of detection by antibodies LM23, BS-400-2, and BS-400-3) and comparatively, xyloglucan contains more side-chains than xylan, which could likely have an effect on the cell wall structure (Meikle et al., 1991; Manabe et al., 2011; Pedersen et al., 2012; Torode et al., 2015). However, negative detection of a polysaccharide is not evidence of its absence, as its presence could be under the level of detection for the method or not expressed in the studied material. The acetylation of the mannan might be an important factor in relation to the types of bindings formed between the mannan and the scaffold polysaccharides. The detected pectin epitope changes over the season and between the extractions supports this idea of a highly flexible matrix.

### Composition and Variation of *Aloe* Polysaccharides

The expectation of finding highly acetylated mannans in at least the *Aloe vera* gel was supported in particular by the binding of CCRC-170, as this antibody has a known target epitope containing acetylations (Pattathil et al., 2012; Zhang et al., 2014). The antibody BS-400-4 bound most strongly to the *Aloe* extractions indicating that the tissue contains high amounts of loosely bound (1→4)-β-D-mannan. We observed a complete lack of binding from the mannan-specific antibody LM22 compared to the binding seen for LM21. Both epitopes was shown to bind mannans by Marcus et al. (2010), although their study was focused on mannan derived oligosaccharides from *Amorphophallus cognac* K. Koch and *Seratonia siliqua* L., which may be structurally different from *Aloe* derived mannans. In particular LM22 has been reported to bind strongly to galactomannan, whereas LM21 binds to glucomannan and the lack of binding of LM21 may therefore suggest glucomannan is not present in *Aloe* (Marcus et al., 2010). The acetylation of mannan has been a concern with regards to the mannan recognizing antibodies as previous studies using the same set of antibodies failed to show any binding (Ahl et al., 2018). In the present study, however, the method has been optimized, especially in terms of sample wait-time, meaning the polysaccharides printed on the nitrocellulose were of a better quality. If samples sit for too long between extraction and printing, signals are likely to fade or even disappear, and a similar situation is expected if samples are frozen (personal observation).

Based on the carbohydrate detecting microarrays results, seasonal variation was detected in the quantity of polysaccharides. The monthly variation was subtle, but when data was pooled according to season, distinct differences were seen. The changes were primarily seen in the binding patterns of pectin and mannan-specific antibodies. Variation in cell wall composition is well-known and reflects the flexibility of the cell wall (Albersheim, 2011). When all data for each species was pooled per season as shown in Figure 2 the changes were not as obvious as when the three extractions steps were compared separately. There was a clear trend, however, of the plants having the highest amounts of polysaccharides in June, July, and August. The optimal harvest time for obtaining higher yield of the sought-after polysaccharides might change from location to location. The ability to detect change in mannan content could therefore be of importance to the *Aloe* industry for planning of harvest time in plantations.

### Potential of Carbohydrate Detecting Microarrays for Authentication of *Aloe* Products

Acetylated mannan from *Aloe vera* has been linked to induced tissue repair in humans for decades (Reynolds and Dweck, 1999; Xing et al., 2014; Thunyakitpisal et al., 2017), but traditionally more than 25% of the genus *Aloe species* (about 150 taxa) have been used to treat a range of conditions (Grace et al., 2009). The primary aim of this study was to investigate if carbohydrate detecting microarrays could be used as a complementary method to detect a seasonal variation in the polysaccharide composition of the selected aloes – two medicinally used and two non-medicinally used. Based on our results, each species had a distinct polysaccharide profile, yet the two medicinally used species (*A. vera* and *A. arborescens*) were more similar to each other than they were to the two non-medicinally used species in terms of both pectin and to some extent mannans. Although, it was possible to differentiate between the *Aloe* species based on carbohydrate detecting microarrays analyses of the investigated samples, the profiles are distinct enough to use carbohydrate detecting microarrays to discriminate unambiguously between individual species. Furthermore, the observed seasonal variation supports the stand that carbohydrate detecting microarrays should not be used as a stand-alone means of analysis to authenticate an *Aloe vera* product. Additional replicate samples would also be needed to explore potential within species variation. However, the similarities and differences between the polysaccharide compositions of the medicinally used species and the non-medicinally ones may potentially be useful to identify the group of medicinal aloes.

As carbohydrate detecting microarrays are primarily a qualitative method, they cannot be used for quantitative authentication of products, but could be suitable to detect if a product actually contains polysaccharides in a composition that could be related to *Aloe vera* or another medicinally used species. The most important antibodies to use for such a screen would be both the mannan-specific ones from which we saw signals in this study, but also pectin-specific antibodies, which showed more differentiation of species. In terms of feasibility for authentication of *Aloe* polysaccharides, carbohydrate detecting microarrays is a high-throughput method capable of simultaneously analyzing 10–20 different samples. Apart from the investment in a microarrayer, the running costs include non-specialist lab equipment, nitrocellulose for printing, and a few selected antibodies. For the analysis of fewer samples, a commercial services analysis of freeze-dried and milled samples could possibly be set up with laboratories having the set up in house. One additional concern is the availability of relevant standardized reference material for comparison. Aloes are rich in phenolic compounds, which can bind and lead to masking of epitopes, and it is therefore important to only use inner leaf mesophyll for the carbohydrate detecting microarray analysis. The commercial plant material of *Aloe vera* and *A. ferox* obtained here as Ph. Eur. reference standard is whole above ground plant material, whereas the monographs specify either exudate or inner leaf mesophyll depending on intended use and the whole plant extracts included here also differed in polysaccharide composition and content (Figure 2 and Supplementary Material). Consequently, there is a need for a more specific definition of what should be considered standard Ph. Eur. reference material.

## Conclusion

The histological micrographs showed differences between species in terms of the amounts of mannan present in different parts of the aloe leaf tissue. The micrographs revealed that the polysaccharides serve as structural hemicellulose in the cell wall but can also function as a storage polysaccharide within the cytosol of *Aloe* species. In terms of Quality Control and Standardization of Plant Based Medicines – carbohydrate detecting microarrays were able to detect differences between species and seasonal variation in composition and abundance of polysaccharides and relevant antibodies are available to screen for the acetylated mannans hypothesized to be responsible for the acclaimed bioactivities of *Aloe* gel. Carbohydrate detecting microarrays therefore has potential as a complementary screening method directly targeting the presence and composition of relevant polysaccharides. The observed seasonal variation may be of importance for commercial growing to optimize harvest times. In addition to seasonal variation, we would expect potential variation of polysaccharides according to the age and origin of the plants, as well as the impact of the local growing conditions, and of storage conditions further down the production line. The carbohydrate detecting microarrays method could thus be used to provide relevant information about variation of the polysaccharides in individual plantations allowing optimization of yield.

## Author Contributions

LA designed the study together with NR and OG, conducted the probing with the antibodies, supervised NA-H and SA-H together with DS and NR, and wrote the manuscript together with NR. LA, NA-H, and SA-H collected and prepared the samples. NA-H and SA-H conducted the carbohydrate detecting microarrays extractions and printing under guidance of LA. LA and BJ conducted the data analysis. JM conducted the microscopy imaging and interpreted the results together with LA. All authors contributed to the discussions and to the final version of the manuscript.

## Conflict of Interest Statement

The authors declare that the research was conducted in the absence of any commercial or financial relationships that could be construed as a potential conflict of interest.
