# Supplementary material for: Detection of Seasonal Variation in Aloe Polysaccharides Using Carbohydrate Detecting Microarrays
Source: Front Plant Sci. 2019 May 14;10:512. doi: 10.3389/fpls.2019.00512 (PMC6527838; doi:10.3389/fpls.2019.00512)
Supplement: Supplementary file 1 [file Table_1.pdf]

Supplementary material to:

Louise Isager Ahl, Narjes Al-Husseini, Sara Al-Helle, Dan Staerk, Olwen M. Grace, William G.T. Willats, Jozef Mravec, Bodil Jørgensen, and Nina Rønsted

Detection of Seasonal Variation in *Aloe* Polysaccharides using Carbohydrate Microarray Polymer Profiling

Frontiers in Plant Science.

Supplementary Material

Heatmap including all species, all seasons, all serial extracts, and all antibodies. The highest mean value of the entire dataset was assigned the value of 100%, and the remainder of the data were adjusted accordingly and normalized with a 5% cut off (represented with a zero - "0").

			Pectin								Mannan								Hemi-celluloses				Secondary AB control			
			Homogalacturonan						RGI																	
			Low methylation	High methylation	Partially methylation				(1→4)-β-D-galactan	(1→5)-α-L-arabinan	(1→4)-β-D-mannan/galactomannan/glucomanna	(1→4)-β-D-mannan/galactomannan/glucomanna	(1→4)-β-D-mannan	Highly acetylated mannan	(1→4)-β-D-mannan/galactomannan/glucomanna	(1→4)-β-D-mannan/galactomannan/glucomanna	(1→4)-β-D-mannan	Highly acetylated mannan	Xylan	XG	(1→3)-β-D-glucan	(1→3)(1→4)-β-D-glucan				
		Antibody	JIM5	JIM7	LM18	LM19	LM19	LM20	LM5	LM6	LM21	LM22	BS-400-4	CCRC-170	LM21	LM22	BS-400-4	CCRC-170	LM23	LM25	BS-400-2	BS-400-3	R	M	R	M
		Array treatment	None	None	None	None	NaOH	None	None	None	None	None	None	None	NaOH	NaOH	NaOH	NaOH	None	None	None	None	None	None	NaOH	NaOH
Water	Aloe Arborescens	Spring	0	0	0	0	0	0	0	0	18	0	56	49	23	0	55	46	0	0	0	0	0	0	0	0
	Aloe Arborescens	Summer	0	0	6	12	12	0	0	0	27	0	77	65	35	0	73	61	0	6	0	0	0	0	0	0
	Aloe Arborescens	Fall	0	0	13	17	19	0	0	0	23	0	71	59	34	0	66	57	0	0	0	0	0	0	0	0
	Aloe Arborescens	Winter	0	0	0	6	7	0	0	0	20	0	54	35	30	0	48	36	0	15	0	0	0	0	0	0
CDTA	Aloe Arborescens	Spring	0	6	27	31	33	0	6	0	13	0	57	51	17	0	55	45	0	0	0	0	0	0	0	0
	Aloe Arborescens	Summer	7	13	33	37	38	8	14	0	18	0	66	55	25	0	63	52	0	10	0	0	0	0	0	0
	Aloe Arborescens	Fall	0	0	19	24	22	0	0	0	14	0	49	31	22	0	49	30	0	18	0	0	0	0	0	0
	Aloe Arborescens	Winter	0	7	21	25	23	0	0	0	16	0	57	50	20	0	55	44	0	6	0	0	0	0	0	0
NaOH	Aloe Arborescens	Spring	0	0	0	0	0	0	0	0	12	0	28	0	18	6	25	0	0	35	0	0	0	0	0	0
	Aloe Arborescens	Summer	0	0	0	0	0	0	0	0	11	0	23	0	16	0	22	0	0	36	0	0	0	0	0	0
	Aloe Arborescens	Fall	0	0	0	0	0	0	0	0	10	0	35	18	15	0	34	16	0	23	0	0	0	0	0	0
	Aloe Arborescens	Winter	0	0	10	11	13	0	0	0	10	0	38	22	16	0	36	19	0	25	0	0	0	0	0	0
Water	Aloe Decaryi	Spring	0	0	0	0	0	0	0	0	24	0	57	46	31	0	56	45	0	0	0	0	0	0	0	0
	Aloe Decaryi	Summer	0	0	0	0	0	0	0	0	32	0	65	50	40	0	59	50	0	0	0	0	0	0	0	0
	Aloe Decaryi	Fall	0	0	8	11	13	0	0	0	23	0	57	45	28	0	52	45	0	0	0	0	0	0	0	0
	Aloe Decaryi	Winter	0	0	0	0	0	0	0	0	23	0	53	25	30	0	48	26	0	13	0	0	0	0	0	0
CDTA	Aloe Decaryi	Spring	0	0	18	22	22	0	0	0	15	0	57	47	21	0	54	48	0	0	0	0	0	0	0	0
	Aloe Decaryi	Summer	0	0	20	25	24	0	0	0	29	0	67	56	36	0	66	52	0	0	0	0	0	0	0	0
	Aloe Decaryi	Fall	0	0	13	17	14	0	0	0	21	0	56	31	29	0	55	30	0	14	0	0	0	0	0	0
	Aloe Decaryi	Winter	0	0	11	16	13	0	0	0	18	0	57	47	22	0	57	43	0	0	0	0	0	0	0	0
NaOH	Aloe Decaryi	Spring	0	0	0	0	0	0	0	0	25	0	48	0	35	7	43	0	0	37	0	0	0	0	0	0
	Aloe Decaryi	Summer	0	0	0	0	0	0	0	0	23	0	43	0	31	0	39	0	0	37	0	0	0	0	0	0
	Aloe Decaryi	Fall	0	0	0	0	0	0	0	0	23	0	49	18	31	0	46	17	0	25	0	0	0	0	0	0
	Aloe Decaryi	Winter	0	0	0	8	10	0	0	0	26	0	58	22	34	0	52	19	0	23	0	0	0	0	0	0
Water	Aloe Vaombe	Spring	0	5	30	33	31	0	8	0	27	0	69	49	36	0	66	51	0	6	0	0	0	0	0	0
	Aloe Vaombe	Summer	9	15	33	36	33	12	0	0	28	0	66	48	37	0	62	49	0	10	0	0	0	0	0	0
	Aloe Vaombe	Fall	0	7	20	22	19	8	0	0	25	0	55	32	35	0	50	32	0	21	0	0	0	0	0	0
	Aloe Vaombe	Winter	0	0	17	22	19	0	5	0	28	0	68	53	35	0	64	50	0	0	0	0	0	0	0	0
CDTA	Aloe Vaombe	Spring	0	0	0	0	0	0	0	0	17	0	33	0	25	0	31	0	0	41	0	0	0	0	0	0
	Aloe Vaombe	Summer	0	0	0	0	0	0	0	0	17	0	32	0	23	0	30	0	0	42	0	0	0	0	0	0
	Aloe Vaombe	Fall	0	0	0	0	0	0	0	0	22	0	44	19	29	0	42	18	0	28	0	0	0	0	0	0
	Aloe Vaombe	Winter	0	0	8	11	12	0	0	0	23	0	51	22	30	0	48	20	0	28	0	0	0	0	0	0
NaOH	Aloe Vaombe	Spring	0	0	0	0	0	0	0	0	35	0	73	55	48	0	69	56	0	0	0	0	0	0	0	0
	Aloe Vaombe	Summer	6	6	7	13	13	7	0	0	46	0	83	66	56	0	78	61	0	0	0	0	0	0	0	0
	Aloe Vaombe	Fall	0	6	14	17	19	6	0	0	40	0	79	63	54	0	73	62	0	0	0	0	0	0	0	0
	Aloe Vaombe	Winter	0	0	0	0	0	0	0	0	28	0	59	36	38	0	55	37	0	17	0	0	0	0	0	0
Water	Aloe Vera	Spring	0	14	0	10	9	14	0	0	16	0	49	40	21	0	45	38	0	0	0	0	0	0	0	0
	Aloe Vera	Summer	0	17	6	10	8	21	0	0	18	0	50	40	27	0	49	40	0	0	0	0	0	0	0	0
	Aloe Vera	Fall	0	13	0	0	0	13	0	0	19	0	49	21	24	0	47	23	0	12	0	0	0	0	0	0
	Aloe Vera	Winter	0	14	0	6	0	13	0	0	20	0	46	34	21	0	42	34	0	0	0	0	0	0	0	0
CDTA	Aloe Vera	Spring	0	0	0	0	0	0	0	0	17	0	38	0	25	6	34	0	0	29	0	0	0	0	0	0
	Aloe Vera	Summer	0	0	0	0	0	0	0	0	27	0	53	0	36	8	47	0	0	31	0	0	0	0	0	0
	Aloe Vera	Fall	0	0	0	0	0	0	0	0	18	0	46	14	25	0	42	13	0	23	0	0	0	0	0	0
	Aloe Vera	Winter	0	0	0	6	7	0	0	0	18	0	48	19	25	0	45	17	0	19	0	0	0	0	0	0
NaOH	Aloe Vera	Spring	0	12	0	0	0	12	0	0	16	0	48	39	21	0	47	37	0	0	0	0	0	0	0	0
	Aloe Vera	Summer	0	15	0	0	0	15	0	0	27	0	61	48	37	0	58	45	0	0	0	0	0	0	0	0
	Aloe Vera	Fall	0	16	0	0	7	16	0	0	17	0	50	41	20	0	46	40	0	0	0	0	0	0	0	0
	Aloe Vera	Winter	0	6	0	0	0	0	0	0	25	0	57	29	32	0	54	27	0	13	0	0	0	0	0	0
Standard	Aloe Vera (std)	Water	0	0	0	0	0	0	0	0	50	0	100	77	65	0	96	78	0	0	0	0	0	0	0	0
Standard	Aloe Vera (std)	CDTA	0	0	23	22	27	0	11	13	32	0	64	40	43	0	63	39	0	19	0	0	0	0	0	0
Standard	Aloe Vera (std)	NaOH	0	0	0	0	0	0	0	0	14	0	32	5	20	0	30	0	0	28	0	0	0	0	0	0
Standard	Aloe Ferox (std)	Water	0	0	12	12	15	0	0	7	17	0	50	38	20	0	51	35	0	0	0	0	0	0	0	0
Standard	Aloe Ferox (std)	CDTA	0	0	0	0	0	0	0	0	0	0	11	5	0	0	12	0	0	0	0	0	0	0	0	0
Standard	Aloe Ferox (std)	NaOH	0	0	0	0	0	0	0	0	0	0	9	0	0	0	9	0	0	0	0	0	0	0	0	0
